# Malignant Syphilis in a Human Immunodeficient Virus-Infected Patient

**DOI:** 10.4269/ajtmh.16-0755

**Published:** 2017-03-08

**Authors:** Sandra Delgado, Jaime Caceres

**Affiliations:** 1 Department of Tropical Medicine and Dermatology, Institute Alexander von Humbolt, Universidad Peruana Cayetano Heredia, Perú.; 2 Department of Pathology, Hospital Cayetano Heredia, Lima, Perú.

Herein, we describe a 25-year-old man who presented a rare widespread form of secondary syphilis characterized by fever and disseminated ulcerative crusted lesions of 1 month duration and in whom malignant syphilis was diagnosed. This is one of the few reported cases of malignant syphilis in adults with human immunodeficient virus (HIV) infection.

**Figure 1.**
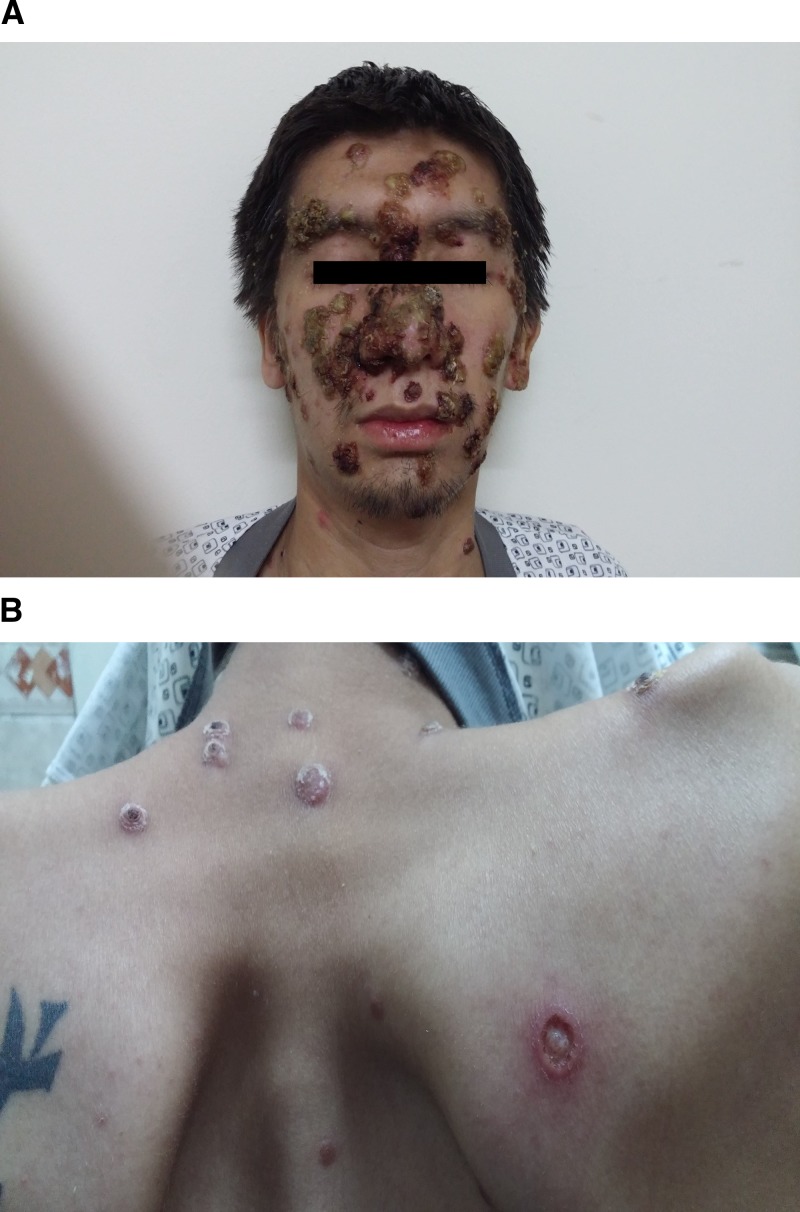
(**A**) Ulcerative crusted lesions can be seen on the face, with involvement of forehead, nose, cheeks and chin associated to purulent draining secretion. (**B**) Papules with ulceronecrotic center on the chest.

**Figure 2.**
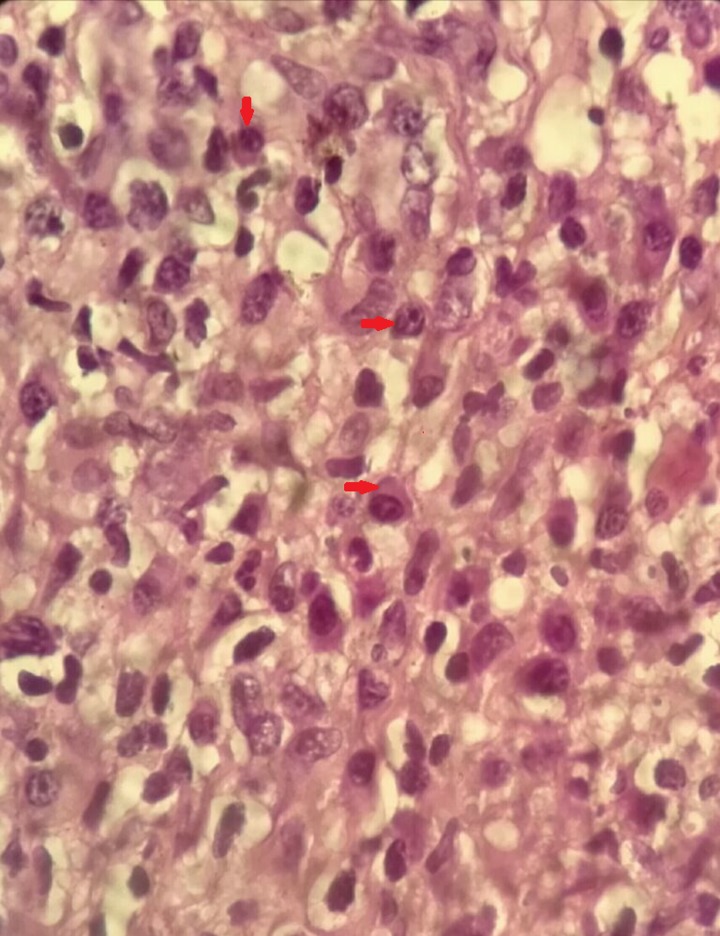
Skin biopsy stained with hematoxylin and eosin. Chronic, granulomatous, non-caseating infiltrate with plasma cells in papillary dermis (magnification ×1,000).

A 25-year-old man was hospitalized with a clinical picture of disseminated ulcerative crusted lesions and fever of 1 month duration. He had a history of papules in the face associated to fever and coryza. Three days before admission, the erythematous papules disseminated to the thorax, upper and lower extremities. The papules progressed to ulceronecrotic lesions associated with purulent discharge (Figure 1A and B

). HIV test and fluorescent treponemal absorption antibodies test were positive; the rapid plasma reagin test was positive at 1:128. CD4^+^ count was 108/mm^3^. A biopsy of the lesion was performed and revealed a chronic, granulomatous, noncaseating infiltrate with plasma cells in the papillary dermis (Figure 2

). The patient was treated with benzathine penicillin and the lesions improved.

Few cases of malignant syphilis had been described in HIV-infected patients. The disease is characterized by fever and a papulopustular eruption that rapidly transforms into necrotic ulcers.^1^ In a multicenter German study involving 11,368 patients with HIV infection, 151 (1.3%) had syphilis but only 11 of them (7.3%) developed secondary malignant lesions.^2^ Often, malignant syphilis^3^ occurs in patients with a CD4^+^ count > 200 cells/mm^3^.^3^ Diagnosis is confirmed by biopsy, being infrequent the identification of spirochetes.^4^ Penicillin is the treatment of choice.
